# A case of membranous nephropathy and myeloperoxidase anti-neutrophil cytoplasmic antibody-associated glomerulonephritis

**DOI:** 10.3892/etm.2014.1852

**Published:** 2014-07-17

**Authors:** ZHI-JUAN HU, KAI NIU, BING LIU, YA-NAN SHI

**Affiliations:** Department of Nephrology, Hebei General Hospital, Shijiazhuang, Hebei 050051, P.R. China

**Keywords:** membranous nephropathy, myeloperoxidase anti-neutrophil cytoplasmic antibody associated glomerulonephritis, crescentic glomerulonephritis, rapidly progressive glomerulonephritis

## Abstract

Membranous nephropathy (MN) may be a primary disease or secondary to autoimmune conditions such as systemic lupus erythematosus, infection (for example, with hepatitis B or C virus), cancer or drugs. In primary MN, crescents are rarely observed. Therefore, the presence of crescents suggests another underlying disease, for example lupus nephritis, anti-glomerular basement membrane disease or anti-neutrophil cytoplasmic antibody-associated glomerulonephritis (ANCA-GN). The coexistence of primary MN and ANCA-GN is rare. In the present case, a 51-year-old female with mild edema in the lower extremities for 1 year was admitted to hospital for renal biopsy. The serum test for myeloperoxidase (MPO)-ANCA was positive. The patient was diagnosed with stage 2 MN with crescentic glomerulonephritis type 3; however, no causal association was found between these two diseases in this case. Treatment was initiated with 500 mg methylprednisolone for 3 days followed by 40 mg of oral methylprednisolone together with 50 mg cyclophosphamide twice per day. One month following treatment, the biochemical data results of the patient had improved.

## Introduction

Membranous nephropathy (MN) is the most common cause of nephritic syndrome in adults, and the disease is usually found in younger individuals. MN may be a primary disease or secondary to autoimmune conditions such as systemic lupus erythematosus, infection (for example, with hepatitis B or C virus), cancer or drugs (1). In primary MN, crescents are rarely observed, and their presence suggests that the patient has another underlying disease, for example lupus nephritis, anti-glomerular basement membrane (GBM) disease or anti-neutrophil cytoplasmic antibody-associated glomerulonephritis (ANCA-GN) (2). Cellular crescents are indicators of severe inflammatory reactions in glomeruli and have been identified in several forms of glomerulonephritis (3). A recent study has shown that apart from inflammatory cells, intrinsic glomerular epithelial cells such as parietal epithelial cells of Bowman’s capsule and glomerular epithelial cells or podocytes contribute to the development of these crescents (4).

In present study, the case of a female patient with MN accompanied by myeloperoxidase (MPO)-ANCA is presented and the possible mechanisms of pathogenesis are discussed.

## Case report

A 51-year-old female with mild edema in the lower extremities for 1 year was admitted to the Department of Nephrology of Hubei General Hospital (Shijiazhuang, China). The study has been approved by the Institutional Ethics Committee of Hebei General Hospital (Hebei, China). Written informed consent was obtained from the patient. The patient had been diagnosed 1 year earlier with hypertension and was treated with amlodipine. In addition, proteinuria had been detected 1 year previously; however, the renal function was unclear. Two days prior to admission, the patient had a urinalysis, which showed protein 3+, occult blood 2+ and a 24-h urine protein of 5.28 g. The patient had no history of cough, shortness of breath, fever, fatigue, nausea, vomiting, weight-loss, decreased appetite or rash.

On admission the patient had a body temperature of 36.4°C, a pulse rate of 60 beats/min, a respiratory rate of 19 breaths/min and a blood pressure of 140/85 mmHg. Mild edema was present in the lower extremities. The results of the complete blood count were as follows: erythrocyte count, 3.73×10^12^/l; hemoglobin, 114 g/l; white blood count, 4.23×10^9^ cells/l; and platelet count, 301×10^9^ platelets/l. The results of urinalysis were: pH 6.5, glucose negative, protein 3+ and occult blood 3+. Examination of the urinary sediment showed 199.2 erythrocytes per high-power field. The test for Bence-Jones protein was negative. Biochemical data were as follows: total protein, 48.0 g/l; albumin, 24.5 g/l; blood urea, 6.62 mmol/l (18.5 mg/dl); creatinine, 123.0 μmol/l (1.4 mg/dl); uric acid, 301.0 μmol/l; total cholesterol, 8.00 mmol/l; total triglyceride, 1.99 mmol/l; high-density lipoprotein, 1.61 mmol/l; low-density lipoprotein, 5.99 mmol/l; very-low-density lipoprotein, 0.9 mmol/l; and glucose, 5.41 mmol/l. The plasma fibrinogen level was 4.12 g/l. The erythrocyte sedimentation rate was 45 mm/h. A test for anti-phospholipid antibody-immunoglobulin (Ig)M was negative. The rheumatoid factor level was 23.4 IU/ml, the antinuclear antibody titer was 1:100 and the C-reactive protein level was 3.0 mg/l. Levels of Ig and complements were found to be normal. The anti-GBM antibody test was negative, the myeloperoxidase anti-neutrophil cytoplasmic antibody (MPO-ANCA) concentration was 62 U/ml (normal range, <10 U/ml), and the serum level of anti-phospholipase A2 receptor antibody was 16.4 μg/ml. The 24-h urine protein was 11.0 g. Serology tests, including tests for hepatitis B surface antigen, hepatitis C virus and HIV were all negative.

The results of the chest X-ray were normal. An ultrasound with parenchyma echo enhancement was performed and the kidneys were found to be of normal size.

**Figure 1:**
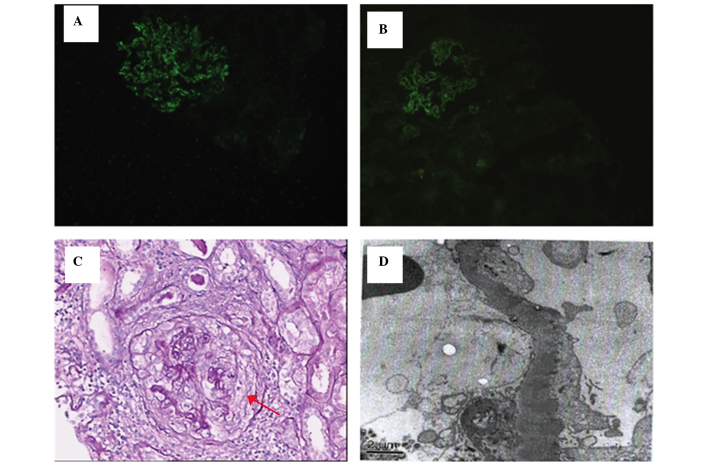
Immunofluorescence microscopy showed granular deposition of (A) immunoglobulin G4 2+ and (B) complement C3 1+ along the glomerular capillary walls (magnification, ×100). (C) Light microscopic examination of a renal biopsy specimen, showing a cellular crescent. Periodic acid-Schiff’s reagent staining (magnification, ×10). (D) Electron microscopy shows an electron-dense deposit in the subepithelial area of the glomerular basement membrane (magnification, ×5,000).

A renal biopsy was performed. Light microscopic examination of the renal specimen containing 31 glomeruli showed characteristic features of MN, with a segmental thickened capillary wall and frequent holes observed using periodic acid-silver methenamine staining. In addition, the 31 glomeruli revealed global sclerosis in 1 glomeruli, cellular crescents in 11 glomeruli, fibrocellular crescents in 7 glomeruli and segmental fibrinoid necrosis of the capillary loop in 1 glomerulus. Tubular atrophy of 50% with interstitial mononuclear cell infiltration and fibrosis was observed. The interlobular artery walls were thick (Fig. 1C). Immunofluorescence microscopy revealed the granular deposition of IgG 3+ and complement C3 1+ along the glomerular capillary walls. IgG subclass analysis showed only IgG4 2+. No staining was observed for IgA, IgM or complements C4 or C1q (Fig. 1 A and B). Electron microscopy revealed an electron-dense deposit in the subepithelial area of the GBM (Fig. 1D). The final diagnosis was crescentic glomerulonephritis (PNCGN) with membranous nephropathy stage 2.

Treatment was initiated with methylprednisolone 500 mg for 3 days followed by oral methylprednisolone 40 mg together with cyclophosphamide (CY) 50 mg twice per day. One month following treatment, the biochemical data of the patient had improved. The results were as follows: total protein, 54.4 g/l; albumin, 33.4 g/l; blood urea, 7.17 mmol/l (20.1 mg/dl); creatinine, 120.0 μmol/l (1.36 mg/dl); and uric acid, 298.0 μmol/l. The 24-h urine protein was 4.29 g, and the MPO-ANCA concentration was 38 U/ml.

## Discussion

ANCA-GN is typically characterized by glomerular necrosis and crescent formation in the absence of significant intracapillary proliferation and without the deposition of Ig and complement. Therefore, it is classified as a pauci-immune type of GN (5). Patients with pauci-immune necrotizing and PNCGN typically present with rapidly progressive glomerulonephritis (RPGN), non-nephrotic range proteinuria and an active urine sediment with red blood cell casts (6,7). PNCGN is an aggressive disease with a 1-year mortality rate of up to 80% in the absence of immunosuppressive therapy. However, the prognosis of PNCGN is significantly improved following immunosuppressive regimens that include corticosteroids and CY therapy.

The coexistence of primary MN and ANCA-GN is a rare, with only a small number of previous cases having been reported (8–15). However, it is not known whether this is a coincidence or if there is a causal association. In the largest clinical study to date investigating MN and ANCA-associated necrotizing and crescentic glomerulonephritis (NCGN), Nasr *et al* (14) found that dual glomerulopathy was a result of the coincidental occurrence of two separate disease processes (14). MN is associated with a greater degree of proteinuria, which has been shown to have a negative impact on the prognosis of this condition. It has been suggested that the diagnosis of MN with ANCA-associated NCGN should be considered in patients who present with RPGN and nephrotic syndrome (14). Hamamura *et al* (16) found that MPO and IgG are partially colocalized within the electron-dense deposits, and demonstrated that MPO-ANCA-GN may lead to MN-like lesions (16). Furthermore, IgG subclass analysis has revealed IgG1 and IgG4 deposition in several MN with ANCA-associated NCGN cases, while IgG4 has been observed in idiopathic MN (13,16). The serum subclass of MPO-ANCA has been found to consist mainly of IgG1 and IgG4 (17).

In the present case, MN and MPO-ANCA-GN were observed simultaneously, and the renal function was normal at biopsy. Immunofluorescence showed granular deposition of IgG and C3 along the glomerular capillary walls, and IgG subclass analysis was positive only for IgG4. In addition, electron microscopy revealed an electron-dense deposit in the subepithelial area of the GBM. In combination, these results indicate an idiopathic MN. The percentage of glomeruli with cellular crescents was 58. In the study by Nasr *et a*l (14), end-stage renal failure (ESRD) was correlated with higher levels of serum creatinine at biopsy. However, no correlation was observed between ESRD and the percentage of glomeruli with cellular crescents or necrosis, or the percentage of open glomeruli, features known to affect the outcome in ANCA-associated NCGN. This was suggested to be due to the small sample size (14). In the present study, the patient was treated with methylprednisolone and CY therapy, and responded well to treatment.

In conclusion, crescents are rare in MN, and usually indicate the presence of another underlying disease, for example lupus nephritis, or a separate disease, for example MPO-ANCA-GN and anti-GBM. The combination may have a causal association. All possibilities, including cancer, drugs and infection associated MN, should be considered prior to the diagnosis of idiopathic MN. Furthermore, the analysis of IgG subclass in the glomerular deposit and the examination of anti-phospholipase A2 receptor antibody levels may be of help in the diagnosis of primary MN.
